# Social media problematic use, memory concerns, and confidence in young female students from an underrepresented student population

**DOI:** 10.3389/fpsyg.2026.1816605

**Published:** 2026-06-10

**Authors:** Maryam BoJulaia, Maura Pilotti

**Affiliations:** Department of Sciences and Human Studies, Prince Mohammad bin Fahd University, Al-Khobar, Saudi Arabia

**Keywords:** media problematic use, memory concerns, Middle East, self-efficacy, undergraduate students

## Abstract

The present study focused on an underrepresented population of female undergraduate students from Saudi Arabia, a society that has only recently given women and men equal rights in education and employment. The country's economic sustainable development plan has made young women's educational attainment a critical constituent of its envisioned success. Yet, social media use among the youth has increased exponentially, leading to concerns. The study examined, through a mixed-methods approach, subject variables that might be differentially linked to academic attainment: social media use (as measured by the Bergen Social Media Addiction scale), self-efficacy (as measured by the General Self-Efficacy questionnaire), prospective and retrospective memory concerns (as measured by the Prospective and Retrospective Memory questionnaire). In regression analyses, problematic use of social media was associated with poor grade point average (GPA). More modestly, GPA decreased with retrospective memory concerns and increased with self-efficacy. Female undergraduate students held ostensibly contradictory beliefs on the relationship between social media usage and academic attainment. They viewed social media tools as useful rather than harmful to academic attainment or memory functioning, and even capable of broadening their self-efficacy. Conversely, they labeled the distractions that social media introduced in their lives as difficult to control. These findings suggest that workshops illustrating evidence of cognitive functioning under conditions of distraction may prevent students from minimizing the costs of problematic social media use.

## 1 Introduction

Sometimes, a research endeavor starts with a recurring concern heard in the corridors of one's workplace. A case in point is the present study, which was initiated by educators' unease about a growing number of undergraduate students who checked their cell phones or laptops for social media communications before, during, and after class time. This frequent and, at times, inappropriate use of social media platforms was often accompanied by the question of whether the observed behaviors would qualify as problematic use. In the extant literature, social media problematic use is defined as “being excessively concerned, overly preoccupied with social media, and spending considerable time and energy on social media that it interferes with an individual's ability to engage in social activities, form interpersonal relationships, study or work, or maintain health and wellbeing” (Bottaro et al., 2025; p. 2; Andreassen and Pallesen, 2014). This broad definition of problematic social media use can be broken down into a set of criteria (Griffiths, 2005) for assessment and treatment purposes: salience (i.e., the use of social media becomes the most important activity in a person's life), tolerance (i.e., the activity demands increasing amounts of time to achieve prior outcomes), mood modification (i.e., social media use is a way to deal with undesirable mental states), relapse (i.e., the activity is no longer under control, thereby leading the user to overindulge in consumption), withdrawal (i.e., unpleasant mental states and physical symptoms are experienced by the user if the consumption of social media is curtailed or blocked), and conflict within oneself and other people. As with other disordered behaviors, manifestations spread over a continuum. Yet, in diagnostic tools, the cutoff point that separates disordered and non-disordered behaviors and mental states is not an agreed-upon matter. For instance, the Bergen Social Media Addiction Scale (BSMAS; Andreassen and Pallesen, 2014; Bottaro et al., 2025), which is a popular measure of disordered behavior and mental states involving social media, does not have a uniform cutoff point. BSMAS contains six statements, each reflecting one of the key components of problematic use (i.e., salience, mood modification, tolerance, withdrawal, conflict, and relapse; Griffiths, 2005), to be rated on a 5-point Likert scale, thereby leading to a range of scores from 6 to 30. In young adults, Luo et al. (2021) report a cut-off score of 24 (range: 6–30), whereas Bányai et al. (2017) prefer 19.

Of course, the most relevant matter to educators is not so much the existence of a uniform cutoff point, which is primarily of interest to clinicians, but rather whether increased usage of social media for non-academic purposes (i.e., off-task use) is linked to declines in academic attainment. The evidence is mostly, but not uniformly, supportive of the notion that social media usage is negatively linked to academic performance. For instance, Alhusban et al. (2022) reported that in a mixed-gender sample of 277 undergraduate students from Saudi Arabia, Jordan, and the United Arab Emirates, social media use was inversely related to GPA. Specifically, low GPA was linked to frequent use of social media applications during class meetings and at night, which interfered with students' ability to prepare for academic activities and function in class. No gender differences were uncovered. Alnjadat et al. (2019), who surveyed a sample of 328 medical students in Saudi Arabia, also reported an inverse relationship but one modulated by gender. Namely, females‘ poor academic performance was more strongly associated with the usage of social media than that of males. Earlier, Walsh et al. (2013) surveyed a sample of 483 female students in the United States. They found women's media use during their first year of college to be inversely related to their academic performance. Instead, Alwagait et al. (2015) did not find a relationship between GPA and hours per week of social media use in a sample of 108 Saudi Arabian students whose gender was not reported. Lau (2017) focused on social media multitasking, which typically involves a user's simultaneous participation in activities, at least one of which is a social-media tool. In a sample of 348 undergraduate students from Hong Kong, Lau found social media multitasking to be inversely related to GPA.

Although most studies are correlational in nature, theoretical frameworks have attempted to explain this inverse relationship by focusing on the user's cognitive processing, either broadly or narrowly defined. The time-displacement hypothesis (Huston et al., 1999) merely suggests that the attractiveness of social media tools, coupled with their perceptual salience and low cognitive demands, may take time and attention away from less appealing and more demanding activities, such as studying or attending class. Cognitive load theory is more precise in its purported effects of social media use. It focuses on the finite attentional resources that the user possesses in working memory, which is the system that is responsible for processing information in one's consciousness. When social media tools are used during class time or while studying or completing an academic task outside the classroom, they divide students' attentional resources between course-relevant and course-irrelevant tasks. An instance of dual-tasking would be reading a textbook chapter on a computer screen or listening to a lecture on an unfamiliar topic (intrinsic load) while monitoring WhatsApp messages on one's cellphone to quickly respond to them (extraneous load). Continuous partial attention to more than one task not only reduces or even depletes one's finite attentional resources but also tends to result in performance declines (e.g., lower or impaired learning; Wood and Zivcakova, 2015). Supporting this conclusion are several studies. For instance, Wood et al. (2012) examined the performance of university students attending a series of lectures, each followed by a quiz on its content. Multitasking was operationally defined as engaging in a social communication activity (e.g., cell phone texting) during lectures. Wood et al. found that non-multitaskers outperformed low, medium, and high multitaskers, suggesting that even minimal use can be distracting. Similarly, Rosen et al. (2013) found that students who engage in social media use during studying had lower GPAs than those who did not.

One of the cognitive functions that may be impaired by distractions is memory. Among the many different types and subtypes of memory processing, two emerge as relevant here: prospective memory (i.e., remembering things to do in the future) and retrospective memory (i.e., remembering things that happened in the past). Specifically, prospective memory is a person's ability to preserve intentions in memory to allow performance of the intended acts at the right time (McDaniel and Einstein, 2007). It may entail mundane tasks, such as remembering to send an email, or essential tasks, such as remembering to take a medication or attend a class meeting. As such, prospective memory plays a critical role in structuring a student's time and behavior. In contrast, retrospective memory refers to a person's ability to recall information from the past, which is stored in long-term memory. It may entail mundane tasks, such as remembering the name of a new classmate, or essential tasks, such as remembering studied concepts during a test. These memory types are not entirely independent. Prospective memory failures may result from not recognizing a cue or a time as a signal to perform a particular action. In contrast, retrospective memory failures may result from being unable to retrieve the actual cue, time, or action from long-term memory. Yet, the consequences of failure are more severe for the former than the latter. Retrospective memory failures are generally attributed to an individual's unreliable memory, thereby being largely overlooked or forgiven, whereas prospective memory failures are attributed to the individual's being unreliable (Munsat, 1965).

Barton and Smyth (2025) found that prospective memory was negatively affected by rapid attention switching, a type of multitasking. Burgess et al. (2000) demonstrated that multitasking relied on both prospective and retrospective memory. Also, Basso et al. (2023) reported that academic performance was predicted by prospective memory functioning. In a separate study, Rakhmanov and Dane (2020) found evidence of the same pattern for retrospective memory functioning. Of course, memory can be assessed directly through testing or indirectly through self-reports. Self-reports measure metamemory, which reflects how individuals assess their prospective and retrospective memory processes and ensuing performance (Csábi et al., 2025). That is, metamemory entails self-evaluations of memory functioning based on similar past experiences and the unique characteristics and encoding circumstances of the events or actions to be remembered (Cauvin et al., 2019). It is considered a key aspect of students' self-regulation of learning, which is critical to academic performance (Balashov et al., 2018; Kancharla et al., 2023). Metamemory reports are deemed to be fairly accurate predictors of memory performance (Kliegel and Jäger, 2006; Melo et al., 2021). A popular tool to assess memory concerns is the Prospective and Retrospective Memory Questionnaire (PRMQ). Its metamemory reports have been found to correspond to actual prospective and retrospective memory test outcomes (Basso et al., 2023; Schnitzspahn et al., 2011), but evidence to the contrary also exists (Sugden et al., 2022; Uttl and Kibreab, 2011). Conservatively, memory concerns gathered through PRMQ may be viewed as measures of self-awareness of one's cognitive functioning in daily activities, such as those involved in determining a student's academic performance. Whether such memory concerns are specifically linked to academic performance is an unclear matter. Waked et al. (2025) found GPA to be inversely related to retrospective memory concerns, but not prospective memory concerns, whereas Pilotti et al. (2025) did not find evidence linking it to either prospective or retrospective memory concerns. Instead, Underwood (2023) reported an inverse relationship. More broadly, Uttl et al. (2018) found that both crystallized and fluid intelligence measures correlated with prospective as well as retrospective measures.

In contrast to memory concerns, which have an unpalatable valence, a desirable subject variable often linked to academic performance is the determination to “get things done” even when obstacles are encountered. Self-efficacy, which reflects this determination, refers to the confidence students possess in their abilities (Bandura, 2001). Self-efficacy is often portrayed as linked to learners' commitment, effort, and persistence, and to fewer reports of negative emotional states during learning (e.g., Travis et al., 2020). Evidence exists that enhancing effort and displaying persistence are the likely responses to challenges by learners with high self-efficacy. Instead, avoidance is the likely response of learners with low self-efficacy (Alhadabi and Karpinski, 2020). Self-efficacy beliefs reflect a sense of agency. As such, high-self-efficacy learners tend to attribute less-than-desirable outcomes to events that can be controlled (e.g., effort). In contrast, low-self-efficacy learners tend to attribute such outcomes to personal ineptitude (Stajkovic and Sommer, 2000).

Self-efficacy is often reported to be positively related to students' engagement (Doo and Bonk, 2020) and academic performance (Bouih et al., 2021; Honicke and Broadbent, 2016; Tossavainen et al., 2021; van Zyl et al., 2022). However, when self-efficacy fosters overconfidence, it may reduce learners' exerted effort (Al Kuhayli et al., 2019; Pilotti et al., 2021). Thus, negative or null correlations have also been found between self-efficacy beliefs and academic attainment. Yet, when self-efficacy is measured in the context of social media, evidence exists that self-efficacy is inversely related to social media use (Nazir et al., 2025). There are two ways in which social media usage may be negatively linked to self-efficacy. One involves attentional processes for task execution, whereas the other pertains to the information that is being made available to the user. If multitasking, such as continuous partial attention to more than one task, is accompanied by a diminished sense of control and self-regulation, self-efficacy, an essential component of self-regulation, may also decline. In addition, exposure to social media messages portraying standards that may be difficult to match by the user (e.g., communications that heighten perceptions of helplessness) may also be linked to declines in self-efficacy (Chen et al., 2025).

## 2 The present study

The present study focused on a population of female undergraduate students in Saudi Arabia, a country that has only recently granted women and men equal legal rights in education and employment (Alhawsawi and Jawhar, 2023). For these women, educational opportunities equal to those of men have symbolized agency and independence. Yet, legal changes have been ushered in by economic necessities. For the Saudi Arabian society at large, young women who are pursuing an undergraduate degree are the indispensable workforce of Vision 2030, a plan to drastically restructure the country's economy to ensure sustainability (Alessa et al., 2022). Thus, these women are under pressure to succeed in their educational endeavors.

In Saudi Arabia as well as around the world, the number of young social media users has increased exponentially over time (Wojdan et al., 2020; Xuan and Amat, 2020). Overall, Saudi Arabian residents spend approximately 7 h per day online, of which 3 h are on social media platforms (AlHamad and AlAmri, 2021). Consequently, the likelihood of excessive use is a widespread concern. Understanding the variables that are linked to young Saudi Arabian women's academic successes and failures is critical to developing interventions that promote their educational and professional attainment. Yet, women remain an understudied population.

The present research examined, through self-reports, subject variables that might be differentially linked to the academic attainment of young Saudi Arabian women, including variables that could be viewed as either strengths (e.g., self-efficacy) or weaknesses (e.g., social media use and memory concerns). In a world currently dominated by technology, the study specifically examined social media use, broadly defined, to determine the extent to which it would be linked to female students' GPA at the end of the semester in which they were surveyed. Memory concerns, measured by PRMQ scores, indexed students' awareness of information processing difficulties that could potentially impair their performance. Self-efficacy scores, gathered from the General Self-Efficacy Questionnaire (GSEQ) of Chen et al. (2001), indexed awareness of their abilities “to get things done.” The following hypotheses were tested:

H1: We predicted that subject variables such as PRMQ and GSEQ scores would be differentially linked to BSMAS scores. Namely, as BSMAS scores increased, awareness of information processing difficulties (metamemory) was expected to increase too (a positive correlation). In contrast, as BSMAS scores increased, reports of self-confidence were predicted to decrease (a negative correlation). The strength of each correlation would indicate the extent to which students were aware of their mental condition, broadly defined as confidence in their abilities (self-efficacy) or narrowly defined as information processing difficulties (PRMQ).

H2: We also predicted that individually BSMAS and PRMQ scores would be inversely related to GPA, whereas GSEQ scores would be positively related to it (H2a). However, when examined together, these subject variables were thought to be differentially linked to GPA. Specifically, we expected BSMAS scores to share a larger portion of the variance in GPA than the other subject variables (H2b). This prediction was based on evidence suggesting that there are costs associated with frequent social media use (Alhusban et al., 2022), despite students' widespread beliefs that there are no downsides (Roberts and Foehr, 2008).

## 3 Method

### 3.1 Participants

The present study involved the recruitment of 456 female undergraduate students enrolled in two compulsory general education courses offered during the first year of university enrollment. Purposive sampling targeted courses, which were specifically selected for focusing on developing critical thinking skills, either generally or in the particular domain of science. These courses were thought to offer students tools and opportunities for self-reflection, making the current study consistent with the university's curriculum learning outcomes. Participants' ages ranged from 18 to 28 years (*M* = 19.90; *SD* = 1.55). There were 243 students pursuing Science, Technology, Engineering, and Mathematics (STEM) degrees (i.e., engineering, computer science, and architecture) and 213 students pursuing non-STEM degrees (i.e., business, law, and design). Participants were enrolled at an English-medium university in Saudi Arabia. Thus, they were bilingual speakers with Arabic as their first language and English as their second language. The minimum sample size for a population of 801 students enrolled in the selected courses was deemed to be 362 with a confidence level of 99%, a margin of error of 5%, and an estimated heterogeneity of the population of 50% (Bartlett et al., 2001; Taherdoost, 2017). The participation rate was 97%.

### 3.2 Materials and procedure

The present study entailed a mixed-method approach to data collection: a quantitative approach via questionnaire administration, and a qualitative approach via individual interviews, and focus group interviews. During the second part of the fall semester of 2025, students were asked to participate in a study on the relationship between actions and mental states. The study involved the online administration of three questionnaires in randomized order. Informed consent, based on the guidelines of the American Psychological Association (APA), was collected before participation.

BSMAS (Andreassen and Pallesen, 2014; Bottaro et al., 2025) was intended to assess students' degree of social media overuse. For the study, social media applications were broadly defined as employing “mobile and web-based technologies to create highly interactive platforms via which individuals share, co-create, discuss, and modify user-generated content” (Kietzmann et al., 2011, p. 241). BSMAS contained six statements (e.g., “How often during the last year have you tried to cut down on the use of social media without success?”), followed by a 5-point Likert scale ranging from “very rarely” (1) to “very often” (5). Each of the six statements reflected established key components of problematic use: salience, mood modification, tolerance, withdrawal, conflict, and relapse (Griffiths, 2005). Together, statements embodied the operational definition of media problematic use as being “excessively concerned, overly preoccupied with social media, and spending considerable time and energy on social media that it interferes with an individual's ability to engage in social activities, form interpersonal relationships, study or work, or maintain health and wellbeing” (Andreassen and Pallesen, 2014; Bottaro et al., 2025, p. 2). Cronbach's Alpha was 0.80.

GSEQ (Chen et al., 2001), which contained eight statements of confidence, was intended to measure students' self-efficacy broadly defined (e.g., “When facing difficult tasks, I am certain that I will accomplish them”). Each statement was to be rated on a 5-point Likert scale ranging from strongly disagree (1) to strongly agree (5) for the extent to which each statement applied to respondents. Cronbach's Alpha was 0.96.

PRMQ (Crawford et al., 2003; p. 275) was administered to assess students' awareness of memory failures in the prospective and retrospective domains. Taken together, statements represented a holistic measure of memory concerns (Blondelle et al., 2020). The 16 statements about memory failures (8 for each memory domain) of the PRMQ were to be rated on a 5-point Likert scale, which included “very often” (5), “quite often” (4), “sometimes” (3), “rarely” (2), and “never” (1). Cronbach's Alpha was 0.89 for both prospective and retrospective scales and 0.94 for all 16 items.

The test-retest reliability of each questionnaire was measured on a sample of 19 participants who took the questionnaires approximately a month apart. The correlations between the scores produced at time 1 and time 2 were above 0.94. Furthermore, before administration, the face validity of the questionnaires was assessed to determine whether they were adequate for the current student population (Nevo, 1985). Four students from the same subject pool independently estimated the extent to which each statement of the BSMAS, GSEQ, and PRMQ measured what it was intended to measure. The constructs tested by the questionnaires were defined and discussed with each rater before statements were rated on a 5-point Likert scale, including “extremely suitable” (5), “very suitable” (4), “adequate” (3), “inadequate” (2), and “irrelevant and therefore unsuitable” (1). BSMAS, GSEQ, and PRMQ statements received a score of either “extremely suitable” or “very suitable”, leading to an interrater agreement of ≥ 0.96.

Each student's GPA acquired during the first year was collected from the Office of the Registrar. It included grades from general education courses compulsory for students across all majors. GPA served as a holistic measure of academic attainment at the university level. As soon as performance records were matched with students' self-reports, any potentially identifying information was deleted from the data set.

Ten students from the same subject pool were also interviewed individually during the second part of the fall semester. These students were asked a set of questions in five key areas: (a) emotional experience (e.g., “How do you usually feel before using social media?”, “How do you usually feel after having used social media?”, and “Do you ever use social media to deal with something in your life?”); (b) academic experience (e.g., “Has your social media use ever affected your ability to study or focus?” and “Can you describe a specific moment when social media helped or harmed your academic performance?”); (c) concentration and mental fatigue (e.g., “Does it take time for your mind to ‘switch back' to studying after scrolling?” and “Do you ever feel mentally drained or overstimulated after being on social media?”); (d) sense of agency (e.g., “Do you feel in control of how much time you spend on social media?” and “Does your use of social media affect your confidence or self-image in any way?”); (e) cultural meaning (e.g., “Do you think social media influences how young people in Saudi Arabia see themselves today?”); and (f) change (e.g., “If you could change one thing about your daily social media habits, what would it be?”).

In addition, 107 students from the same subject pool, organized in small groups of 2–4 individuals, were asked a subset of questions before or after class meetings (abbreviated interviews): (a) emotional experience (e.g., “How do you usually feel before using social media?”, “How do you usually feel after having used social media?”); (b) academic experience (“Can you describe a specific moment when social media helped or harmed your academic performance?”); and (d) sense of agency (e.g., “Do you feel in control of how much time you spend on social media?”). An additional question examined students' views of the potential relationships between memory concerns and use of social media (“Does using social media before or during academic tasks help or harm your ability to remember information you studied or something you need to do in the near future, such as attend a meeting with your advisor?”).

Students were interviewed by a trained research assistant. Interview notes were submitted to thematic analysis (Braun and Clarke, 2021) to identify and categorize the underpinning themes. Specifically, the guidelines of the coding reliability approach of Braun and Clarke (2021) were followed. A theme included remarks that were mentioned by at least 50% of the participants. To analyze the interview data, a two-step process was used. Two independent raters who had expertise in social and behavioral science research examined students' responses. The inter-rater reliability score was 92%. If coding discrepancies between the two raters existed, they were addressed by a third rater.

As noted in the introductory section, the present study was motivated by faculty who expressed concerns about social media use in the classroom. Thus, before the collection of students' data was initiated, 19 faculty teaching the selected student population were informally asked to report the frequency with which they observed at least one student texting during class meetings on a 4-point scale, including “not at all”, “once or twice a week”, “many times a week”, and “nearly every day I teach”. They were also asked to indicate the extent to which this behavior was widespread among students on a 4-point scale, including “none”, “a few”, “many”, and “almost all”.

The University where the study was conducted (PMU) follows a US curriculum and mode of instruction. Thus, the data-collection process was approved by the Deanship of Research to comply with the guidelines of the Office for Human Research Protections of the U.S. Department of Health and Human Services (PMU-DoR-2024-2025-28). Informed consent, which was obtained from all participants, was deemed by the Deanship of Research to conform to the APA ethical guidelines for the treatment of research participants. The study complied with the Strengthening The Reporting of OBservational Studies in Epidemiology (STROBE) guidelines for cross-sectional designs (see Mehta and Naveed, 2025).

## 4 Results

### 4.1 Questionnaire responses

Data were analyzed through JASP Team (2026). Table 1 displays the descriptive statistics of students' responses to the BSMAS, GSEQ, and PRMQ, along with first-year GPA. Inferential statistics were carried out to determine the degree to which social media use, self-efficacy, and prospective and retrospective memory concerns were related. Results were considered significant at *p* < 0.05. In adherence to evidence suggesting that prospective memory failures are more salient and costlier than retrospective memory failures (Munsat, 1965; Pilotti et al., 2025), students reported more prospective concerns [*t*_(455)_ = 49.53, *p* < 0.001].

**Table 1:** Mean *(M)* and standard deviation *(SD)*.

Variables	Range	*M*	*SD*
BSMAS	1–5	2.98	0.84
SE	1–5	3.84	0.81
Prospective memory	1–5	3.58	0.74
Retrospective memory	1–5	2.58	0.85
GPA	0–4	3.03	0.54

Table 2 displays significant zero-order correlations between the subject variables selected for this study. H1 predicted that as BSMAS scores increased, awareness of information processing difficulties (metamemory) would increase too (a positive correlation), whereas reports of self-confidence would decrease (a negative correlation). H1 was supported. However, the strength of each correlation was thought to offer an insight into the extent to which students were aware of their mental state (either broadly defined as self-confidence, GSEQ scores, or narrowly defined as information processing difficulties, PRMQ scores) related to social media use. Coefficients of determination were rather modest, accounting for no more than 10% of the variance in BSMAS scores.

**Table 2:** Zero-order correlations.

Variables	BSMAS	SE	Prospective memory	Retrospective memory
SE	−0.20			
Prospective memory	+0.31	−0.38		
Retrospective memory	+0.22	−0.35	+0.86	
GPA	−0.52	+0.28	−0.29	−0.26

Correlations are significant at *p* < 0.05.

H2a predicted that BSMAS and PRMQ scores would be inversely related to GPA (serving as a holistic index of performance), whereas GSEQ scores would be positively related to it. To test this prediction, zero-order correlations were carried out, which simply indicated the strength and the direction of individual binary relationships without consideration of the other subject variables. In these analyses, GPA was found to be inversely related to social media use (as measured by BSMAS) and prospective and retrospective memory concerns (as measured by PRMQ). Instead, GSEQ scores increased with GPA. H2a was supported.

H2b predicted that when considered simultaneously, these subject variables would be differentially associated with GPA. Specifically, BSMAS scores would exhibit a stronger link with GPA than the other subject variables. To determine the relative strength of the links between participants' self-reports and GPA, linear regression analyses were carried out (see Table 3). These analyses were performed separately for prospective and retrospective memory concerns due to their elevated zero-order correlation. H2b was supported. GSEQ scores were positively related to GPA, whereas social media use (as measured by BSMAS) and retrospective memory concerns were inversely related to it. Yet, the negative relationship between social media use and GPA was far superior to that of retrospective memory concerns and GPA. Prospective memory concerns failed to be significantly related to GPA when the other subject variables (i.e., GSEQ and BSMAS scores) were considered, even though students reported more of these concerns.

**Table 3:** Regression analyses with BSMAS, SE, and either prospective memory concerns or retrospective memory concerns as the predictors and GPA as the outcome variable.

Variables	*b*	*SE b*	*Beta*	*p*	*Part*
Constant	3.797	0.183			
BSMAS ^*^	−0.299	0.026	−0.468	< 0.001	−0.44
SE ^*^	0.090	0.025	0.152	< 0.001	+0.14
Prospective Memory Concerns	−0.061	0.032	−0.084	= 0.056	−0.08
Constant	3.779	0.157			
BSMAS ^*^	−0.301	0.026	−0.471	< 0.000	−0.46
SE ^*^	0.086	0.025	0.145	= 0.001	+0.14
Retrospective Memory Concerns ^*^	−0.070	0.027	−0.109	= 0.010	−0.10

First panel: *R*^2^ = 0.31 Second panel: *R*^2^ = 0.32; Tolerance ≥ 0.91. * = significant contribution to GPA.

When BSMAS, PRMQ, and GSEQ scores were viewed as different indices of students' metacognition, evidence pointed to metacognition as being weakly related to performance, except for BSMAS. Furthermore, when the responses to the BSMAS statement that specifically focused on the impact of social media use on performance (i.e., “You use social media so much that it has had a negative impact on your job/studies”) were considered, only 31% selected ‘often' or ‘very often'. The remaining 69% selected ‘sometimes', ‘rarely', or ‘very rarely'. The responses to this statement were correlated with GPA (*r* = −0.40, *n* = 456, *p* < 0.001). However, only 16% of the variance in the responses to the statement accounted for GPA.

Students' awareness of the impact of a problematic behavior on academic attainment could be considered the first step toward change. The results of quantitative analyses suggested limited students' metacognition, and thus limited room for change. Furthermore, to equate the scales of different questionnaires, responses to the BSMAS were averaged rather than summed, obscuring the cut-off score to identify individuals who suffered from social media problematic use. When summed responses were computed (range: 6–30), further support for the link between disordered use of social media and academic attainment emerged. Specifically, if the cut-off point of 24 was selected (Luo et al., 2021), only 14% of the 456 young female participants reached that score. These individuals displayed an average GPA of 2.54 (*SD* = 0.57) compared with the rest of the sample, whose average GPA was 3.11 (*SD* = 0.49). However, if the cut-off of 19 points was adopted (Bányai et al., 2017), 42% of our sample qualified as exhibiting social media problematic use. Their average GPA was 2.74 (*SD* = 0.50) compared with the rest of the sample, whose average GPA was 3.25 (*SD* = 0.46). Not surprisingly, even by adopting a more liberal cut-off point, the GPA of those fitting the problematic use category was lower [*t*_(454)_ = 11.36, *p* < 0.001].

### 4.2 Interview responses

The analyses of students' responses in individual and focus-group interviews served to clarify the results of quantitative analyses. In this section, reports of cause-and-effect relationships or lack thereof are assumptions made by students and collected by the researchers. For instance, most participants admitted that social media (e.g., instant messaging, texting, web browsing, and emailing) wasted their time. However, they denied that it affected their academic performance. Furthermore, when the primary reasons for using social media were reported, an uneven frequency distribution emerged. Mentioned first in 84% of the comments were personal communication motives, including sharing work or daily events with friends and family, as well as planning for and managing activities. Other motives were also mentioned, but rarely in the first place, such as entertainment (10%), as well as learning and general information gathering (6%). Participants noted that they usually used social media following notifications or to counteract boredom. They admitted to checking and responding to messages on applications, such as WhatsApp, Signal, and Telegram, often during class meetings. They justified texting by noting that it was expected of them to respond promptly to family members, friends, and classmates. This behavior was viewed as a temporary distraction with no notable consequences. Students often added that it did not interfere with their ability to participate in class activities. There was a clear-cut difference between applications and their reported setting of autonomous use. Applications such as Instagram, YouTube, TikTok, Snapchat, and Twitter (X) were said to be used preferentially at home, during commuting and other types of travel, and when waiting for the next class. Applications such as WhatsApp, Signal, and Telegram were those that qualified for widespread use, including classrooms. Irrespective of the location, students reported that after use, they often felt overwhelmed by the amount of information they received. Participants spontaneously reported experiences in which the intent to use a social media platform for only a few minutes failed. As a result, they found themselves spending several hours on it. Experiences of this nature were reported to be the main reason for wanting to reduce usage and gain control. Moreover, participants indicated that they often needed a few minutes before being able to resume activities, such as studying.

Notwithstanding concerns, participants had an overall positive view of social media. They identified several advantages, such as being motivated by successful individuals, using social media platforms as sources for studying, completing assignments, sharing information and artifacts, cultivating relationships, and becoming more confident and open-minded. Participants did not report negative effects on their confidence, broadly defined or restricted to academic tasks, or self-image, albeit some experienced these issues at a younger age. The only concern that participants expressed was their struggle to gain greater control over their use of different platforms to optimize the limited free time that academic demands allowed. As noted earlier, students did not report that texting during class would disrupt their attention to activities, as texting was described as a brief, irregular activity that purportedly was an unavoidable necessity (e.g., “During class, I read only important messages”, “It takes only a minute”, “I check only messages from my mom”, “I need to tell my driver when to pick me up”, and “I check notifications on my cellphone for information about schedule changes”). They also acknowledged that accessing platforms on YouTube, TikTok, Snapchat, and Twitter (X) between class meetings was a way to rest. Yet, switching off these platforms to start class meetings was somewhat unpleasant, making class meetings more demanding and less enjoyable, especially if lectures were given.

The reports of instructional faculty on the frequency with which they observed at least one student texting during class meetings consistently pointed to “nearly every day I teach” as the unanimous preferred answer. They also chose “many” or “almost all” to report the extent to which the activity was widespread among students. Faculty repeatedly reminded students that using cell phones during class meetings was distracting, impaired their performance, and was not permitted. Yet, the behavior reoccurred unabated. The themes that were reported by faculty as students' responses to their being caught texting were similar to those expressed by students individually and in focus groups.

## 5 Discussion

The most relevant findings of our investigation were the contradictions that emerged from the data. For instance, in interviews, students reported that they were aware of the distractions that social media introduced into their lives and admitted to their difficulty in controlling the amount and timing of daily consumption. They recognized that switching to off-task behaviors (e.g., checking incoming text messages and responding to them) while studying or completing an assignment was distracting. However, they were reluctant to admit that this type of multitasking could negatively affect their on-task performance or merely slow it. They often noted that off-task behaviors, such as checking text messages, were well-practiced, almost automated tasks. As such, these behaviors would be unlikely to interfere with the on-task activity of studying, listening, reading, or writing either in class or while preparing for classes. On the contrary, off-tasks were a welcome break. Overall, participants held a positive image of social media as a set of useful tools for studying and cultivating relationships. In interviews, some students even denied that social media use might be linked to poor academic attainment. However, quantitative evidence suggested otherwise by illustrating an inverse relationship between GPA (a holistic measure of academic attainment) and evidence of social media use (as measured by BSMAS).

Although our findings did not entail any direct measure of multitasking, the correlation between BSMAS scores and GPA, combined with students' reports of distraction, aligns with the extant literature suggesting multitasking as a likely mediator (e.g., Lau, 2017; Wood et al., 2012; Wood and Zivcakova, 2015). Our findings comply with those of surveys showing that many students believe that there are no costs associated with multitasking (e.g., frequent checking of e-mails and text messages, listening to music or talks, and scanning e-readers) while studying or completing assignments (Roberts and Foehr, 2008), even though evidence exists that frequent task switching rarely occurs without costs. As such, our findings reflect how students deal with the potential cognitive dissonance arising from holding inconsistent cognitions (Jeong et al., 2019). To avoid the discomfort stemming from contradictions, they magnify the advantages and minimize the costs of social media use. Other potential cognitive distortions that may account for the discrepancy between students' subjective perceptions and actual reality may include specific biases in information processing. Optimism bias is one of such distortions, which reflects people's tendency to overestimate the likelihood of positive outcomes and underestimate that of negative outcomes. In this context, students' optimism bias would lead them to overestimate the benefits of social media use and be oblivious to its costs. Among the selected student population, optimism bias, which tends to manifest itself in grade predictions (Pilotti et al., 2024), may also shape evaluations of a multitude of behaviors linked to social media use. Another potential bias is the illusion of control (Nowotny, 2024), which may lead students to overestimate their ability to control the processing of information that is fed to them via text message applications or other social media tools. Yet, compared to the other biases, the illusion of control bias may play a minor role among the selected student population, as students reported that they were aware that the estimated time spent on a social media application often tended to be shorter than the actual time.

Our findings of an inverse relationship between BSMAS scores and GPA agree with those of Alhusban et al. (2022), who reported that social media use was inversely related to the GPA of undergraduate students from Saudi Arabia, Jordan, and the United Arab Emirates, and with those of Alnjadat et al. (2019) and Ketari and Khanum (2013), who collected data in Saudi Arabia from medical students and undergraduate students, respectively. Outside of the Middle East, a similar inverse link between social media use and academic performance was reported by Walsh et al. (2013), who surveyed undergraduate students in the United States, and Lau (2017), who surveyed undergraduate students from Hong Kong. However, our results are inconsistent with those of Alwagait et al. (2015), who did not find a relationship between Saudi Arabian students' GPA and hours per week of social media use. The null finding reported by Alwagait et al., albeit based on a small sample, suggests that the link between academic performance and amount of use is likely to be shaped by the manner of use (e.g., in class while listening to a lecture, at home while studying, or during breaks from on-task activities). Although severe social media use may make the manner of use moot, since the individual's social media habits intrude on all aspects of daily life, it may matter in gray areas that do not qualify as excessive practices but are still substantial. Indeed, the extant literature seems to agree that the key ingredient for an inverse relationship between social media use and academic attainment is the degree of distraction from on-task activities that social media applications introduce into students' already demanding lives. Important to note, however, is that distraction may be difficult to measure, as it may be as overt as that arising from the traditional multitasking setting (e.g., texting while listening to a lecture), or as hidden as that arising from thinking about social media materials while being engaged in an on-task activity.

### 5.1 Implications and applications

Our findings point to Saudi Arabian female undergraduate students as being particularly in need of practical demonstrations showing the consequences of multitasking on cognition. Implementing successful interventions to mitigate disordered social media usage may benefit students' academic attainment if interventions do not simply focus on curtailing use (Radtke et al., 2021) or on specific tasks, such as detecting fake news (e.g., Guess et al., 2020) and preventing cyber threats (e.g., Higdon, 2022; Lee and Hancock, 2023). Self-Determination Theory (SDT; Andersen et al., 2024; Ryan and Deci, 2000) may guide the development and implementation of interventions. According to SDT, a behavior, such as social media use, is engaged and maintained because it satisfies three key human needs: autonomy, competence, and relatedness (i.e., desire to connect with others). This theory provides insights into why students may frequently use social media (West et al., 2023). For instance, using social media may satisfy the need for autonomy in four areas (Festl, 2021): evaluation (e.g., to appraise posts), motivation (e.g., to be recognized), emotion (e.g., to reduce boredom), and creativity (e.g., to post materials to express oneself). The need for competence may also be satisfied if the interaction with a given social media platform gives the user a sense of expertise in how the platform works and can be used. The need for relatedness can be fulfilled through participation (e.g., to post messages and materials), communication (e.g., to discuss issues), learning (e.g., to teach or learn from others how to best use applications), and instances of morality in action (e.g., to practice mutual respect).

The quantitative results of our study suggest that subjective competence (as measured by self-efficacy beliefs) may act as a double-edged sword. On one hand, it may serve as a buffer against the potential negative impacts of students' social media use on daily functioning. On the other hand, it can also be viewed as preventing students from acknowledging such effects. The qualitative results offer a broader view of the role of autonomy, competence, and relatedness as motives for social media use. Among women who have recently emerged from a strict patriarchal order, which constrained their autonomy, social media may represent an emblem of autonomy. However, in interviews, only a few of these young women referred to the past social order and its restrictions. The past was often an afterthought for such women, who were focused on shaping their future. Yet, the present mattered as participants admitted that social media applications satisfied their need for relatedness, including sharing daily life events with their nuclear and extended family, friends, and classmates.

Interventions may focus on autonomy and competence needs as the most likely to be amenable to change. Indeed, when students report that their control of the use of social media is weak, they acknowledge that autonomy needs are unsatisfied. However, when students do not recognize the impact of social media use on academic performance, they fail to notice that their competence needs are unsatisfied. Workshops that engage students in concrete demonstrations of how specific social media activities can detract from on-task activities, such as reading, listening, and writing, may be particularly helpful. Platform-specific digital literacy interventions based on particular core proficiencies, such as those proposed by Ha and Kim (2024); (e.g., understanding of a media environment and its social context, technical capability, and attitudes of the user), may be particularly helpful. Such interventions can teach students to recognize the specific cognitive costs and benefits of different social media types. Yet, it is only by showing students concrete evidence of the consequences of particular behaviors on social media platforms (e.g., multitasking) while performing academic tasks that may increase their awareness of the consequences of their behavior may increase. Particularly helpful may be demonstrations based on the experimental design of Demirbilek and Talan (2018). Demonstrations of strategies to enforce timely disengagement from social media may also be helpful.

### 5.2 Limitations

The limitations of this study include the exclusively female sample, which prevented generalization of findings to male students. Similarly, all participants were undergraduate students and relatively young. Whether the same findings would apply to older students and to other educational levels is to be determined. Extant evidence of individual differences in academic attainment (including demographic factors, personality, cognitive ability, motivational factors, etc.; Richardson et al., 2012) also suggests that unmeasured subject variables might limit the generalizability of the present findings. Self-reports might also have fostered response biases (Kemmelmeier, 2016), such as social desirability bias, deflating BSMAS and PRMQ scores and inflating those of GSEQ, and acquiescence bias, having the opposite effects on BSMAS and PRMQ. Furthermore, the cross-sectional design of the present study did not allow us to infer cause-and-effect relationships between degrees of social media use and academic attainment.

#### 5.2.1 Data about platform-specific mechanisms

The quantitative measures upon which this design relied did not address differences in social media platforms, their use, and potential cognitive impact. For instance, the lack of granularity of BSMAS scores masked the difference between passive scrolling and active communication. In the extant literature, texting, scrolling, clicking, and viewing short-form and long-form videos (e.g., YouTube, TikTok, Snapchat, etc.) are reported to have qualitatively different brain markers and cognitive impacts. For instance, Satani et al. (2025) found that social media use was linked to marked alterations in brainwave activity, with patterns being dependent on the user's behavior. Active interaction was linked to enhanced Beta and Gamma waves, whereas passive surfing was accompanied by enhanced Theta and Delta waves. Cognitive load was indexed by declines in Alpha waves. Purported differences in cognitive processing and learning were reported by Romero and Temena (2025), who proposed that the continuous information intake associated with scrolling is linked to increased cognitive load. The task-switching arising from clicking adds mental strain (depletion of cognitive resources) to an already overloaded mind. Romero and Temena also proposed that the habit of scrolling may be accompanied by memory retention impairments and deficient deep learning, whereas the habit of clicking may be associated with the difficulty that learners experience focusing on a single task for prolonged periods. Cognitive processing differences may even be reflected in the type of display. Chen and Lin (2016) reported that text display types on mobile devices affect cognitive load during reading differently, with static displays yielding lower cognitive load than dynamic displays.

Important to note, though, that although the questionnaire data analyses of our study did not identify platform-specific data, the qualitative analyses of interview responses highlighted that during class times, students intermittently engaged in problematic use of particular platforms (e.g., reading text messages along with texting). Even in this context, a primary limitation of our data was their inability to distinguish between passive scrolling of text messages and active communication. Of course, direct observation of students' spontaneous scrolling, texting, and their likely coexistence in the classroom might be viewed as ethically problematic and unlikely to be able to differentiate these behaviors. On the other hand, under controlled conditions, the impact of passive scrolling and active communication on the cognitive processing of the selected student population could be measured.

#### 5.2.2 Future directions

Notwithstanding its limitations, the current study offered a snapshot of an understudied female student population in an ecosystem dominated by technology and gender-related gains. Participants were intended to represent a slice of the population that the 2030 Vision contemplates to be the female workforce of the new Saudi Arabian economy. Their self-reports, although limited to one point in time, illustrated female students' views of their interactions with social media applications.

Planned research will be devoted to addressing current shortcomings through varied methodological approaches. Under consideration is the use of a longitudinal design to identify trends through the application of a time series analysis to data from self-reports. Although platform-specific tracking is unfeasible due to privacy concerns, a within-subjects experimental design is also under consideration, which aims to assess the impact of multitasking on critical thinking activities. Of primary interest are the cognitive demands of different types of multitasking, such as the degree to which passive scrolling of text messages or active communication may interfere with reading or listening comprehension in a second language.

Understanding students' views and their cognitive functioning in a world defined by a variety of social media platforms is key to identifying the ways in which the human mind adapts to environmental changes. Furthermore, it is also key to developing effective interventions that moderate social media use to promote the academic attainment of women who are the main agents of change in the struggle to reduce archaic gender gaps in education and employment.

## Data Availability

The original contributions presented in the study are included in the article/supplementary material, further inquiries can be directed to the corresponding author.
